# Misleading early blood volume changes obtained using ferumoxytol-based magnetic resonance imaging perfusion in high grade glial neoplasms treated with bevacizumab

**DOI:** 10.1186/s12987-016-0047-9

**Published:** 2016-12-20

**Authors:** Joao Prola Netto, Daniel Schwartz, Csanad Varallyay, Rongwei Fu, Bronwyn Hamilton, Edward A. Neuwelt

**Affiliations:** 1 Department of Neurology, Oregon Health & Science University, 3181 SW Sam Jackson Park Road, Portland, OR 97239 USA; 2 Department of Neuroradiology, Oregon Health & Science University, 3181 SW Sam Jackson Park Road, Portland, OR 97239 USA; 3 Advanced Imaging Research Center, Oregon Health & Science University, 3181 SW Sam Jackson Park Road, Portland, OR 97239 USA; 4 School of Public Health, Oregon Health & Science University, 3181 SW Sam Jackson Park Road, Portland, OR 97239 USA; 5 Emergency Medicine, Oregon Health & Science University, 3181 SW Sam Jackson Park Road, Portland, OR 97239 USA; 6 Department of Veterans Affairs Medical Center, 3710 SW U.S. Veterans Hospital Road, Portland, OR 97239 USA; 7 Department of Neurosurgery, Oregon Health & Science University, 3181 SW Sam Jackson Park Road, L603, Portland, OR 97239 USA

**Keywords:** High grade glioma, Perfusion MRI, Ferumoxytol, Bevacizumab

## Abstract

**Background:**

Neovascularization, a distinguishing trait of high-grade glioma, is a target for anti-angiogenic treatment with bevacizumab (BEV). This study sought to use ferumoxytol-based dynamic susceptibility contrast magnetic resonance imaging (MRI) to clarify perfusion and relative blood volume (rCBV) changes in glioma treated with BEV and to determine potential impact on clinical management.

**Methods:**

16 high grade glioma patients who received BEV following post-chemoradiation radiographic or clinical progression were included. Ferumoxytol-based MRI perfusion measurements were taken before and after BEV. Lesions were defined at each timepoint by gadolinium-based contrast agent (GBCA)-enhancing area. Lesion volume and rCBV were compared pre and post-BEV in the lesion and rCBV “hot spot” (mean of the highest rCBV in a 1.08 cm^2^ area in the enhancing volume), as well as hypoperfused and hyperperfused subvolumes within the GBCA-enhancing lesion.

**Results:**

GBCA-enhancing lesion volumes decreased 39% (P = 0.01) after BEV. Mean rCBV in post-BEV GBCA-enhancing area did not decrease significantly (P = 0.227) but significantly decreased in the hot spot (P = 0.046). Mean and hot spot rCBV decreased (P = 0.039 and 0.007) when post-BEV rCBV was calculated over the pre-BEV GBCA-enhancing area. Hypoperfused pixel count increased from 24% to 38 (P = 0.007) and hyperperfused decreased from 39 to 28% (P = 0.017). Mean rCBV decreased in 7/16 (44%) patients from >1.75 to <1.75, the cutoff for pseudoprogression diagnosis.

**Conclusions:**

Decreased perfusion after BEV significantly alters rCBV measurements when using ferumoxytol. BEV treatment response hinders efforts to differentiate true progression from pseudoprogression using blood volume measurements in malignant glioma, potentially impacting patient diagnosis and management.

## Background

Treatment for newly diagnosed glioblastoma (GBM) consists of resection followed by radiotherapy with concomitant temozolomide (TMZ). Increased enhancement volume on T_1_-weighted magnetic resonance imaging (MRI) using gadolinium-based contrast agent (GBCA) after chemoradiotherapy (CRT) in GBM patients can represent tumor progression or pseudoprogression. Diagnostic accuracy is critical in deciding appropriate therapy. Response assessment in neuro-oncology (RANO) suggests that differentiation of pseudoprogression from disease progression cannot be accomplished with a conventional MRI method in the first 12 weeks post-CRT [1].

Ferumoxytol, an iron oxide nanoparticle, is FDA-approved for iron replacement and used off-label for brain imaging, especially in patients with compromised renal function for whom GBCA is contraindicated [2]. Ferumoxytol as an MRI contrast agent for perfusion benefits from its intravascular property. Compared to GBCA, it may not require leakage correction or preload dosing [3]. Perfusion MRI has been used clinically as a biomarker to prospectively differentiate pseudoprogression from disease progression [4–7], and relative cerebral blood volume (rCBV) measurements using ferumoxytol as an MRI contrast agent can discriminate true progression from pseudoprogression in GBM using a mean rCBV threshold of 1.75 [3, 8]. The use of small paramagnetic iron oxides (SPIOs), such as ferumoxytol, was first published as an MR-based imaging agent for cancer in 1989, and has increased exponentially in the last decade. There were 728 publications on SPIOs and cancer between 1990 and 2010 and more than 864 in the last five years alone.

Bevacizumab (BEV) is an anti-vascular endothelial growth factor (VEGF) A antibody working as an angiogenesis inhibitor [9] that normalizes tumor vasculature [10], and decreases contrast-enhancing tumor volume and blood volume in lesions both in animal models [11, 12] and clinically [13–15]. No association has been found between tumor volume change after BEV and overall or progression-free survival [16]. After treatment with BEV, tumor perfusion decreases but tumor growth may only be temporarily inhibited [17, 18], leading to “pseudoresponse” and mismanaged therapy. Due to the likelihood of pseudoresponse and normalization of the blood–brain barrier, traditional methods to assess post-CRT tumor progression can be misleading in patients treated with BEV [1], and there is a concern that evaluating CBV for differentiating real progression from pseudoprogression as a result of CRT after treatment with BEV may produce false negative results. This study hypothesizes that perfusion decrease in high grade tumors after CRT and subsequent treatment with BEV can affect clinical management, and aimed to determine rCBV changes post-BEV using ferumoxytol-based dynamic susceptibility contrast (DSC) MR perfusion in patients with high grade glioma in a clinical setting.

## Methods

### Data collection

Sixteen patients (mean age in years [range]: 54 [39–63]; 6 males, 53 [44–62]; 10 females, 52 [43–61]) presenting with a total of 21 lesions (5 patients had two distinct and isolated lesions) were included in this retrospective study and provided written informed consent on one of four prospective Oregon Health & Science University HIPAA-compliant and IRB-approved MRI protocols (2753, 2864, 1562, and 813) using two contrast agents, GBCA and ferumoxytol, between 2009 and 2012. On average, pre-BEV MRI studies were performed 7 days (range: 0–21) before drug infusion, and post-treatment MRIs were performed 27 days after (range: 12–30).

Inclusion criteria were that patients have histological evidence of high grade glioma; evidence of increased or new enhancing tumor on clinical scan, which at time of scan could have represented tumor progression or treatment related changes; having received CRT and bevacizumab in the course of disease treatment; and an MRI study with gadoteridol and ferumoxytol DSC MRI less than 21 days before BEV and less than 30 days after. All patients received standard CRT with either 60 Gy in 30 fractions or 59.4 Gy in 33 fractions, with concomitant oral TMZ at a dose of 75 mg/m^2^/day. After completion of CRT all patients continued on monthly TMZ (150–200 mg/m^2^/day for 5 days in every 28 day cycle) for at least 6 months or until disease progression occurred [19].

### Imaging

Patients underwent 3T MRI (Siemens Trio or Philips Achieva) with a multichannel head coil. All imaging protocols included anatomical and dynamic sequences in the axial plane. T_1_-weighted scans (repetition time [TR]/echo time [TE] ≈ 900 ms/10 ms, field of view [FOV] = 240 mm^2^, matrix = 256 × 256, ~44 2 mm slices with no gap) were acquired with a standard dose of 0.1 mmol/kg intravenous (IV) gadoteridol to assess contrast enhancing volume. DSC scans (90 measurements at 0.66 Hz, TR/TE/flip angle = 1500 ms/20 ms/45°, FOV = 192 mm^2^, matrix 64 × 64, 27 3 mm slices with a 0.9 mm gap) were acquired, with a short IV bolus of 1 or 2 mg/kg (~5–10 ml) ferumoxytol injected at a flow rate of 3 ml/s followed by 20 ml of saline flush at the same rate. Approximately 20 measurements were obtained before the contrast reached the brain allowing an accurate baseline signal. Patients did not receive ferumoxytol directly after gadoteridol; in some cases, the two agents and their associated imaging acquisitions were separated by at least 12 min, and in some cases the acquisitions took place during sessions on consecutive days. However, recent work has shown that dual contrast sessions are feasible and yield reliable measurements of rCBV [20].

### Image analysis

Tumor volume measurements were taken as the product of the extent of the GBCA-enhancing areas in three orthogonal axes as measured by a neuroradiologist with 8 years of experience (JPN). All DSC data were processed by JPN using NordicICE. CBV maps were generated by applying a tracer kinetic model to the first pass of the contrast bolus. Voxelwise CBV maps were coregistered to T_1_-weighted images and then normalized by dividing by the mean of normal appearing white matter CBV in the same region in the contralateral hemisphere (rCBV), defined by JPN and chosen in the same axial slice as the lesion when possible, or if not possible, in the most proximal axial slice to the lesion.

One region of interest (ROI) was drawn on each GBCA-enhancing lesion (JPN) pre- (ROI_pre_) and post-BEV (ROI_post_) in each patient. The slice with the largest enhancing area was chosen based on the standard clinical evaluation for brain tumor assessment (RANO criteria) [1]. Care was taken to exclude blood vessels and areas of cystic change or necrosis, defined as abnormal areas with hyperintense signal on T_2_-weighted and hypointense signal on T_1_-weighted without discernible enhancement after GBCA injection.

Mean tumor rCBV, the voxelwise ferumoxytol-based DSC measurement, was calculated over each GBCA-enhancing ROI. Three means were calculated: (1) pre-BEV rCBV over ROI_pre_, (2) post-BEV rCBV over ROI_post_, and (3) post-BEV rCBV over ROI_pre_. The third mean was calculated because BEV caused an expected reduction in the extent of GBCA enhancement, likely due to a reduction in vascular permeability secondary to vascular normalization; however, changes in local blood volume due to BEV likely occur beyond the extent of post-BEV GBCA enhancement.

The mean of “hot spot” (the mean of the highest rCBV in a 1.08 cm^2^ area in the enhancing volume, the default size of a standard circular region of interest in Nordic ICE) rCBV values and pixel-wise rCBV histograms were obtained for each ROI. A standardized (% voxels in each bin/total voxels) histogram was determined using 40 bins 0.25 wide (range: 0–10). The proportion of voxels with rCBV > 1.75 was defined as hyperperfused subvolumes. This threshold value provides the optimal sensitivity and specificity for differentiating low grade from high grade glioma using rCBV [21]; it also has been used to differentiate treatment related changes and true progression using ferumoxytol or leakage-corrected GBCA perfusion MRI [3]. There is no established threshold for delineating hypoperfused subvolumes [15]. rCBV ≤ 0.75 was used, which represents perfusion at least 25% lower than normal appearing contralateral white matter.

A linear mixed effects model was used to compare differences in enhancing volume, mean and hot spot rCBVs, as well as hyperperfused and hypoperfused subvolumes before versus after BEV while taking into the account observations within the same patient. The Dunnett-Hsu method was used to adjust for multiple comparisons with the before BEV period as the control group. Least square (LS) mean ± standard error (SE) are reported. To assess the likelihood of BEV treatment to affect diagnostic criteria and clinical management, the proportion of patients with lesions that changed from high rCBV (≥1.75) before BEV to low rCBV (<1.75) after BEV was also calculated. For patients with two lesions, if both lesions changed from high to low rCBV, or one lesion changed from high to low and the other lesion remained low before versus after BEV, it was counted as a change that would likely impact clinical management. If one lesion changed from high to low and the other lesion remained high, it was not counted as a change that would likely impact clinical management. All analyses were conducted using SAS 9.3 (SAS Institute Inc., Cary, NC, USA.).

## Results

Fourteen patients were diagnosed with GBM, one with anaplastic astrocytoma, and one with anaplastic oligodendroglioma. Patients received BEV due to increasing enhancing lesion without clinical symptoms in 8 (50%) cases, increasing enhancing lesion with clinical symptoms in 7 (44%) cases, and clinical worsening but radiographically stable disease in 1 (6%) case.

**Fig. 1:**
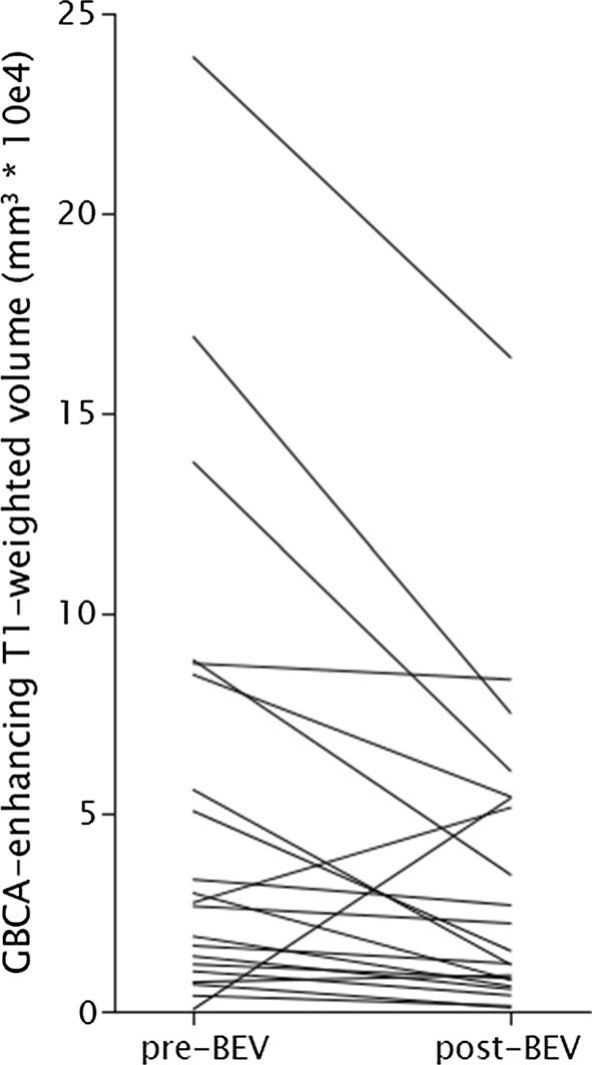
GBCA-enhancing volume on T_1_-weighted MRI significantly decreased (P = 0.005) post-BEV

**Fig. 2:**
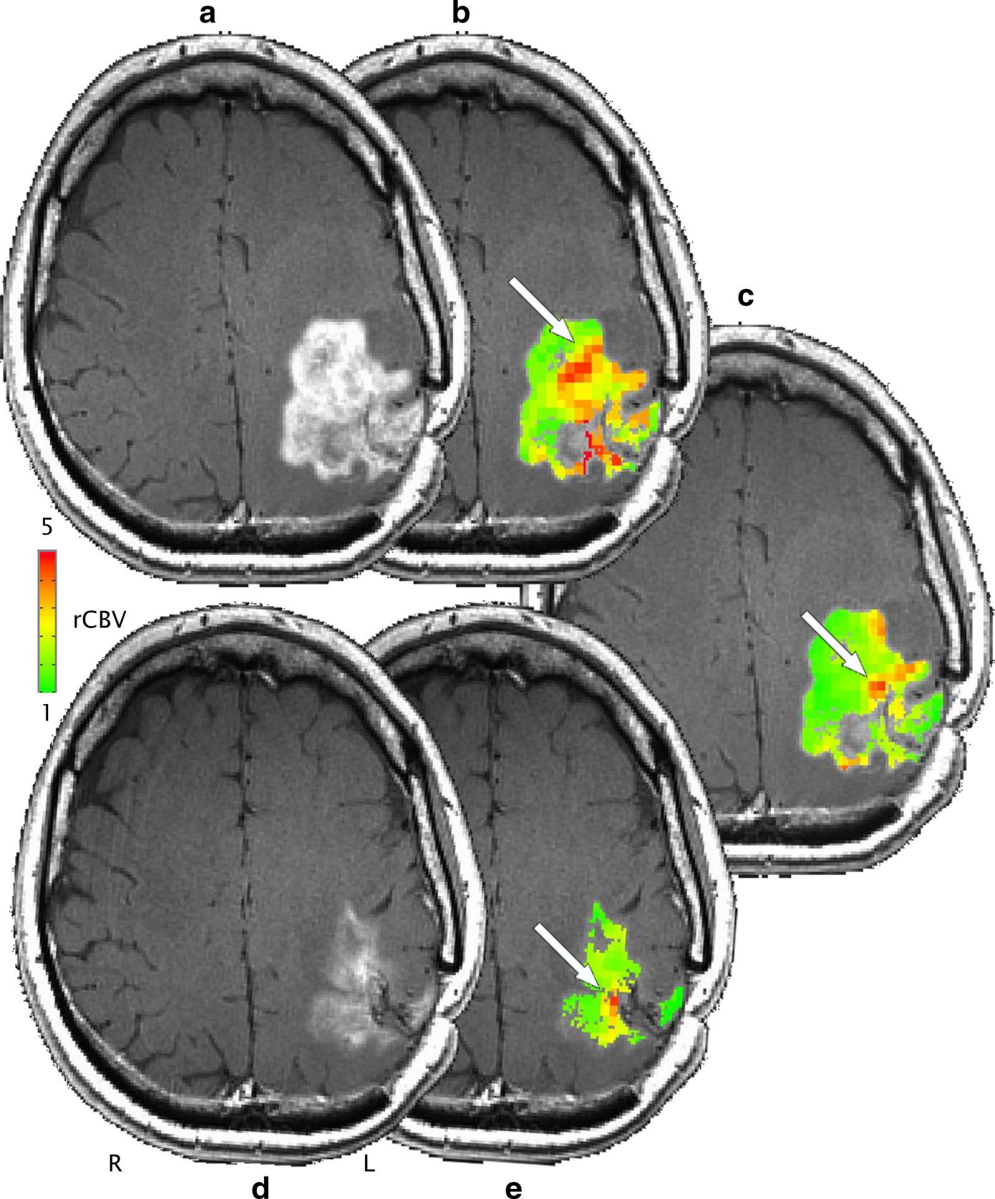
GBM in 44 y/o male: GBCA enhancement on T_1_-weighted pre- (**a**, ROI_pre_) and post-BEV (**d**, ROI_post_). rCBV maps derived from ferumoxytol DSC pre- (**b**) and post-BEV (**e**) overlaid on the enhancing volume. An overlay of the post-BEV rCBV map on ROI_pre_ can be found in **c**. “Hot spot” regions are indicated by *white arrows*; note the marked decrease in rCBV between **b** and **c**

After BEV treatment, 18 out of 21 (86%) GBCA-enhanced T_1_-weighted tumor volumes decreased. The mean volume was significantly smaller post-BEV, decreasing approximately 39% (pre-BEV, 55,807 mm^3^ ± 11,422; post-BEV 34,474 mm^3^ ± 11,422; P = 0.005, Fig. 1, exemplified in patient in Fig. 2).

**Fig. 3:**
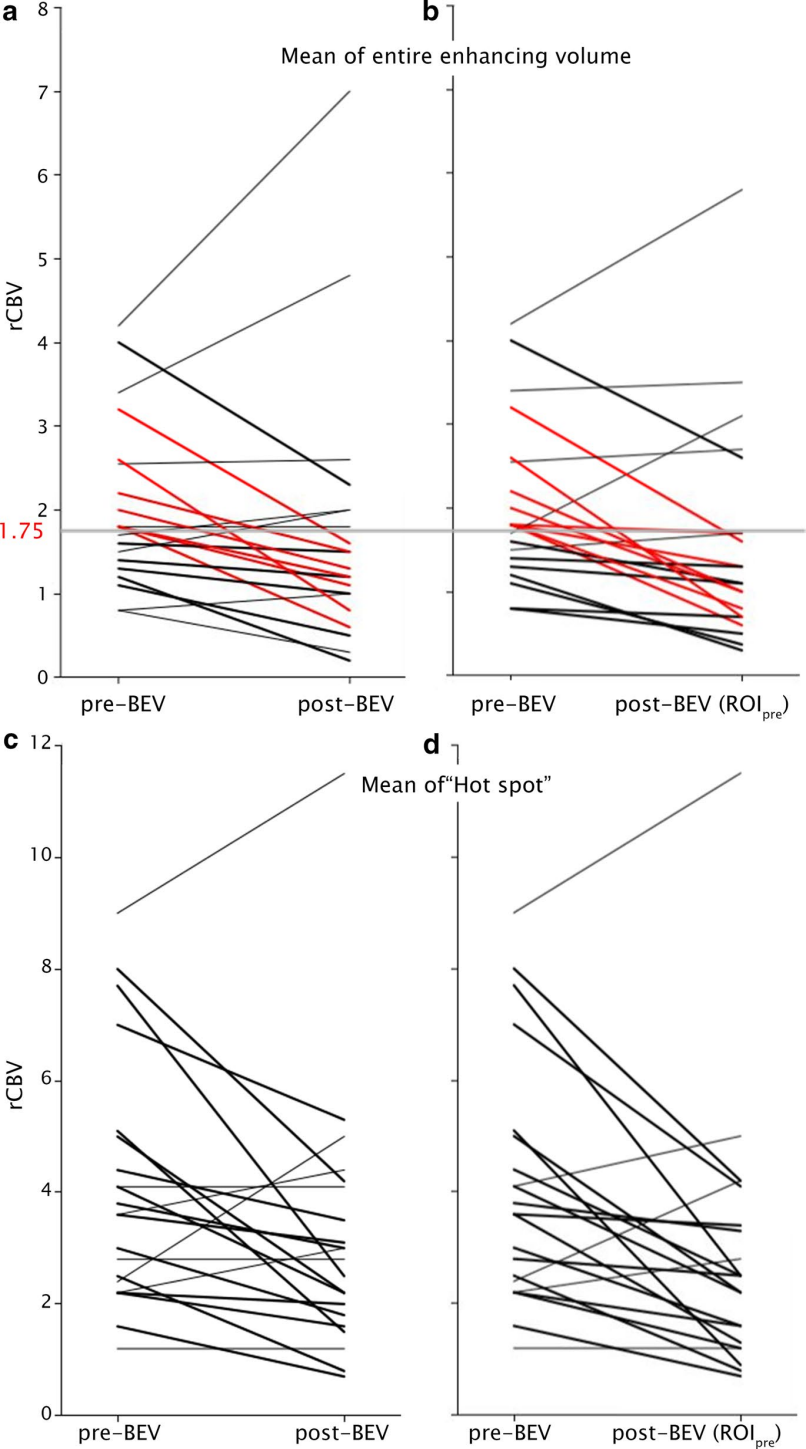
Change in rCBV pre- and post-BEV for each of 21 lesions. A *black line* indicates a decrease in rCBV, *red* indicates a decrease in rCBV that passed the clinically relevant threshold of 1.75, and the remaining lesions are marked in *gray*. Values for the entire enhancing volume are shown in **a** and **b**. **c** and **d** depict hot spot changes. **a** and **c** depict the rCBV change between pre-BEV measured over ROI_pre_ and post-BEV rCBV measured over ROI_post_. **b** and **d** depict the rCBV change between pre-BEV measured in ROI_pre_ and post-BEV rCBV in ROI_pre_

**Fig. 4:**
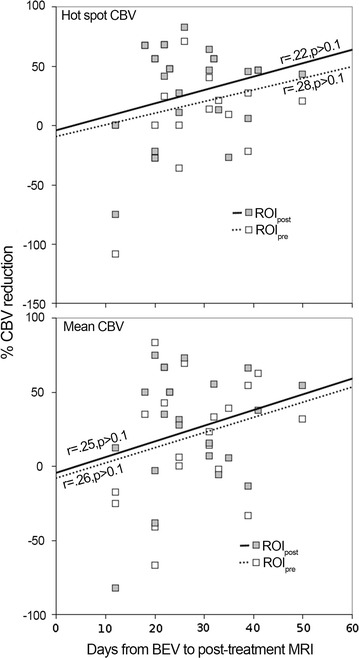
Relationship between rCBV measurements and time interval to post-treatment MRI. *Top* hot spot rCBV measurements in ROIpost (*filled*) and ROIpre (*open*); *y axis* is % rCBV reduction, a negative value indicates rCBV increase. *Bottom* mean rCBV over the entire enhancing lesion. No significant relationships of rCBV with post-treatment MRI time intervals were found (all P > 0.1)

When examining perfusion measurements in each lesion after BEV treatment, there was no significant difference in mean rCBV values in ROI_pre_ vs. ROI_post_ (1.95 ± 0.35–1.65 ± 0.32, respectively, P = 0.227, Fig. 3a). However, mean “hot spot” rCBV decreased between pre- and post-BEV (4.04 ± 0.64–3.13 ± 0.52, respectively, P = 0.046, Fig. 3c). When post-BEV rCBV values averaged over ROI_pre_ were compared to pre-BEV mean rCBV values over ROI_pre_, mean and “hot spot” values decreased to 1.51 ± 0.30 (P = 0.039, Fig. 3b), and 2.81 ± 0.52 (P = 0.007, Fig. 3d), respectively. The relationship between rCBV changes and timing of the scan with respect to BEV administration was tested with Pearson’s r. There were no significant findings in hot spot or mean rCBV analyses (Fig. 4).

For the assessment of the likelihood of BEV treatment to affect diagnostic criteria and clinical management, 7/16 (44%) patients had lesions that changed from high rCBV (≥1.75) to low rCBV (<1.75) when mean pre-BEV rCBV values over ROI_pre_ were compared to mean rCBV post-BEV over ROI_post_ (Fig. 3a: red lines). In addition, 2/16 (13%) patients had lesions that changed from low rCBV (<1.75) to high rCBV (≥1.75). Similarly, when calculating post-BEV rCBV over ROI_pre_, 7/16 (44%) changed from high to low rCBV (Fig. 3b: red lines), and one (6%) changed from low to high rCBV.

**Fig. 5:**
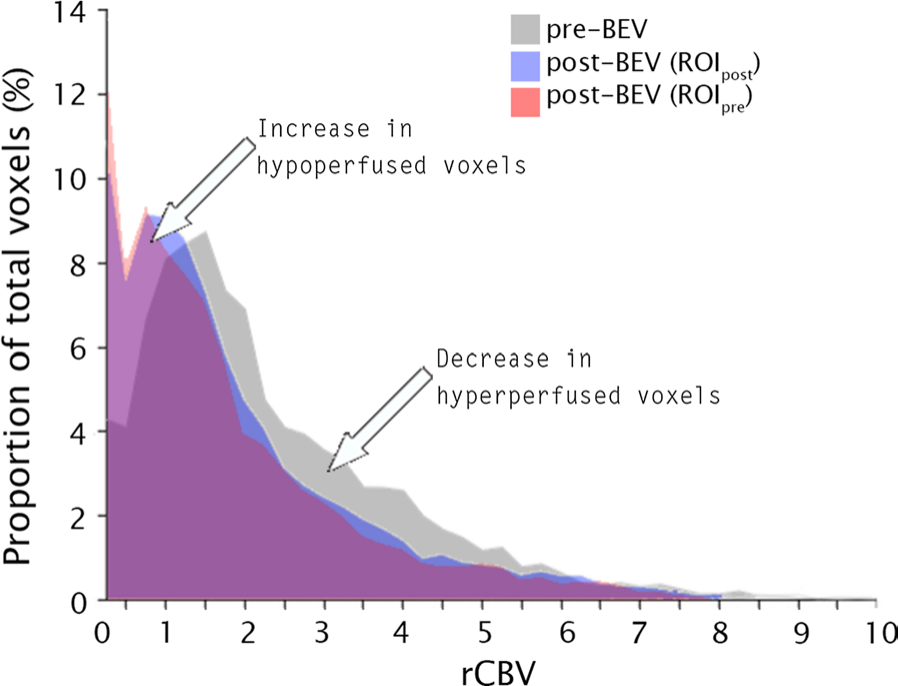
Distribution of the averaged over all patients’ rCBV voxel values before administration of BEV (pre-BEV, *gray*), after it using the pre-BEV volume of enhancement (*red*, ROI_pre_) and post-BEV volume of enhancement (*blue*, ROI_post_). There is a significant increase in the proportion of hypoperfused voxels (P = 0.008) and a significant decrease in hyperperfused voxels (P = 0.01) in both ROIs after BEV administration

More detailed examination of rCBV changes was accomplished through distribution analysis. The average histogram in ROI_post_ vs. ROI_pre_ demonstrated a significant increase in the mean proportion of hypoperfused voxels from 24% pre-BEV to 38% post-BEV (P = 0.0007), and a significant decrease in hyperperfused voxels from 39% pre-BEV to 28% post-BEV (P = 0.017, Fig. 5: gray vs. blue). Similarly, when the histogram of post-BEV rCBV was generated over ROI_pre_, the mean proportion of hypoperfused voxels increased to 41% from pre-BEV (P = 0.0001), and the mean proportion of hyperperfused voxels decreased to 26% (P = 0.006) (Fig. 5: gray vs. red).

## Discussion

This study used ferumoxytol to determine perfusion changes in patients with high grade glioma after treatment with bevacizumab. The results show that after treatment with anti-VEGF agent BEV there is a significant decrease in GBCA-enhancing volume, and a decrease in perfusion using ferumoxytol in the enhancing area of high grade gliomas after chemotherapy and radiation therapy but only when the pre-treatment enhancing regions of interest (ROI_pre_) were used. The use of pre-treatment enhancing regions of interest (ROI_pre_) over which to assess post-BEV perfusion maps increased sensitivity in all cases, suggesting that this method should be considered when quantifying blood volume changes after BEV. However, given the reduction in mass effect known to accompany BEV treatment it is possible that tissue shift could account for the difference. In the histogram analysis, it becomes clear that the decrease in the mean rCBV happens as expected due to BEV treatment, quantified by an increase in hypoperfused and a decrease in hyperperfused subvolumes. Nearly half of lesions changed from high rCBV before the BEV administration to low rCBV post-BEV. This is particularly important for cases in which perfusion MRI will be used to assess new or increasing enhanced areas for possible treatment.

Several publications describe the association between high CBV and tumor progression and low CBV and pseudoprogression and treatment related changes [8, 22, 23]. The ideal cutoff value has been under intense debate [4, 6, 24, 25]. We use 1.75 as a threshold rCBV value at our institution given significant indications that using this threshold for planning subsequent treatment is correlated well with overall survival [3]. Given that a significant percentage of lesions changed from high rCBV before the BEV administration to low rCBV after its infusion (44%), it is important to acquire perfusion MRI before the administration of BEV if rCBV will be used to determine whether a new or increased enhancing lesion represents true progression of disease or changes due to CRT. Other previously published threshold values range from 0.7 to 2.6; had they been utilized in this study, it is possible that the interpretation of the effects of bevacizumab on rCBV as it pertains to criteria for pseudoprogression, may have been different [6, 24, 25]. Extreme threshold values, high or low, would have less impact as the majority of cases having values under or above the threshold before and after bevacizumab administration would increase, respectively.

The RANO treatment response evaluation criteria are based on clinical symptoms and conventional MRI (T_1_-weighted GBCA-based contrast volume enhancement) findings. Treatment related changes and the diagnostic challenge it represents are well known, including the impossibility of reliable evaluation with conventional MRI. A number of advanced imaging techniques have been studied including diffusion, spectroscopy, DCE and DSC perfusion MRI and nuclear medicine techniques, all with variable results. Our group has shown that cerebral blood volume measurements based on DSC perfusion, particularly in cases where a blood pool agent such as ferumoxytol is used, is a reliable method that can be used to differentiate between pseudoprogression and real tumor progression [3]. In 56% of the lesions, there was no significant change in the diagnosis and interpretation of the perfusion values. There are several possible alternatives for this finding. If baseline rCBV was very high before bevacizumab administration and decreased after treatment, it is possible that rCBV values did not go below the threshold, maybe related to aggressive tumor progression with some resistance to the effect of bevacizumab. A floor effect is also possible in areas of tumors that are poorly perfused; if rCBV values were already very low before bevacizumab administration, an eventual decrease in perfusion values will not change the diagnosis of pseudoprogression.

Leakage correction is necessary for accurate quantification of perfusion MRI using GBCA; rCBV measurements using gadoteridol can adequately distinguish between patients with treatment related changes versus true progression only after leakage correction is applied [3]. The confounding effect of BEV and the infrequent use of leakage correction may be two critical factors contributing to the inconsistency in the literature of using rCBV to determine disease progression status [4, 26–28]. Finally, relative perfusion values from ferumoxytol-based DSC in GBM clearly match those in recent reports, without the need for leakage correction or preload dosing as is currently necessary for accurate assessment of tumor perfusion with GBCA [11].

Lastly, recent reports have showed clear region-specific long term deposition of gadolinium in the brain [29]. While it is not yet clear whether there are any adverse health effects of gadolinium accumulation, these reports are serious enough to warrant a FDA safety announcement [30]. Ferumoxytol does not extravasate from the blood pool into brain parenchyma nearly as readily as do most GBCAs used in clinical practice today. While the incidence of serious anaphylaxis risk is approximately 10 times higher using ferumoxytol compared to most GBCAs, the absolute incidence of severe side effects remains small, approximately 1:10,000. Current evidence and experience indicates that ferumoxytol is a safe and available alternative for GBCA for assessment of tumor progression versus CRT-related changes.

Limitations of this report include the choice of a single slice method for pixel-wise histogram analyses. This technique is based on the RANO criteria for assessment of brain tumor in the clinical practice and for clinical trials, but it is possible that important information was missed by not using the entire tumor. It is unlikely that results would change based on the location of the slice or the use of the entire tumor, and single slice measurements are the norm in clinical practice. In addition, it is difficult to precisely control the relative timing of BEV, CRT, and MRI exams. While it is plain that the timing of these exams with respect to treatment phase can have profound effects on results, the cohort in this study was carefully chosen to have as narrow a window of timing variability as possible.

## Conclusions

In conclusion, due to blood volume reduction caused by BEV, the threshold of rCBV > 1.75, used to differentiate high rCBV from low rCBV after CRT, may be confounded early after BEV administration and thwart true diagnoses of vascular progression, true response, or treatment related changes. Ideally, pre-BEV measurements should be used and extreme care taken with clinical interpretation of perfusion MRI results in any patients who have received BEV, even when using an intravascular agent such as ferumoxytol.

## Abbreviations

BEV: bevacizumab
CRT: chemoradiotherapy
DSC: dynamic susceptibility-weighted contrast
GBCA: gadolinium based contrast agent
GBM: glioblastoma multiforme
FOV: field of view
IV: intravenous
JPN: author and experienced radiologist
MRI: magnetic resonance imaging
RANO: response assessment in neuro-oncology
rCBV: relative cerebral blood volume
ROI: region of interest
TE: echo time
TMZ: temozolomide
TR: repetition time
VEGF: vascular endothelial growth factor
